# The Biological Roles of ZKSCAN3 (ZNF306) in the Hallmarks of Cancer: From Mechanisms to Therapeutics

**DOI:** 10.3390/ijms252111532

**Published:** 2024-10-27

**Authors:** Wenfang Li, Han Zhang, Jianxiong Xu, Ayitila Maimaitijiang, Zhengding Su, Zhongxiong Fan, Jinyao Li

**Affiliations:** 1 School of Pharmaceutical Science, Institute of Materia Medica, Xinjiang University, Urumqi 830017, China; 2 Xinjiang Key Laboratory of Biological Resources and Genetic Engineering, College of Life Science and Technology, Xinjiang University, Urumqi 830017, China

**Keywords:** zinc finger protein with KRAB and SCAN domains 3 (ZKSCAN3), ZNF306, zinc finger protein, hallmarks of cancer

## Abstract

ZKSCAN3 (also known as ZNF306) plays a pivotal role in the regulation of various cellular processes that are fundamental to the development of cancer. It has been widely acknowledged as a key contributor to cancer progression, with its overexpression consistently reported in a broad spectrum of malignancies. Importantly, clinical studies have demonstrated a significant association between elevated ZKSCAN3 levels and adverse prognosis, as well as resistance to therapeutic drugs. Specifically, ZKSCAN3 promotes tumor progression by enhancing multiple hallmark features of cancer and promoting the acquisition of cancer-specific phenotypes. These effects manifest as increased tumor cell proliferation, invasion, and metastasis, accompanied by inhibiting tumor cell apoptosis and modulating autophagy. Consequently, ZKSCAN3 emerges as a promising prognostic marker, and targeting its inhibition represents a potential strategy for anti-tumor therapy. In this review, we provide an updated perspective on the role of ZKSCAN3 in governing tumor characteristics and the underlying molecular mechanisms. Furthermore, we underscore the clinical relevance of ZKSCAN3 and its potential implications for tumor prognosis and therapeutic strategies.

## 1. Introduction

A significant impediment to extending life expectancy is the predominance of cancer as the primary global cause of mortality. According to estimates, approximately 20 million new cancer cases and 9.7 million cancer-related deaths were recorded globally in 2022 [1,2]. Although early cancer detection has slowed the incidence rate of cancer, several common cancer types continue to exhibit notable progression [2,3]. Therefore, it is essential to uncover novel prognostic markers and therapeutic targets for effective cancer management.

The development of tumors, as well as the progression from benign tumors to malignant cancers, is an intricate process primarily caused by abnormal gene expressions [4]. Tumor cells exhibit distinctive characteristics known as “cancer hallmarks”, which differentiate them from normal cells [5,6,7]. These hallmarks include sustained proliferative signaling, phenotypic plasticity, deregulated cellular metabolism, resistance to cell death, genome instability and mutation, senescence, vascularization, evasion of growth suppressors, nonmutational epigenetic reprogramming, immune evasion, replicative immortality, tumor-promoting inflammation, polymorphic microbiomes, and invasion-metastasis [3,8]. In addition to mutations in protooncogenes and tumor suppressor genes, dysregulation of transcriptional, translational, post-translational, and epigenetic regulators also contributes significantly to tumorigenesis and malignant transformation [9,10,11,12]. Over the past two decades, numerous studies have revealed that Zinc finger proteins (ZNFs) constitute the largest group of transcriptional factors in mammalian cells [13,14,15,16]. They play a crucial role in the initiation, growth, and progression of cancerous tumors by regulating gene transcription and translation [17,18,19]. Furthermore, their potential involvement in drug resistance suggests that ZNFs could serve as novel biomarkers and therapeutic targets for future interventions [20].

**Figure 1:**
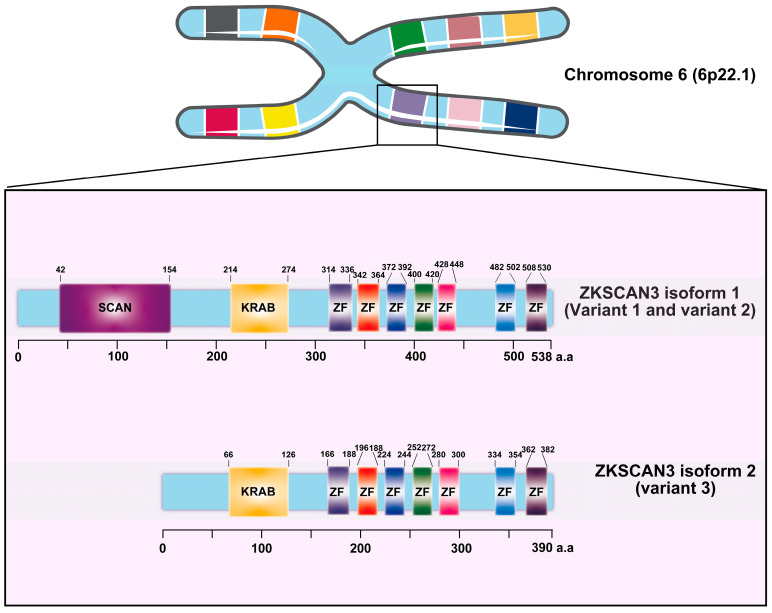
The diagram provided herein depicts the intricate structure of the Krüppel-associated box zinc finger (ZF) proteins, with a specific focus on the ZKSCAN3 factors and their constituent domains. Derived from the comprehensive UniProt database, this representation illustrates the organization of ZKSCAN3, which comprises a KRAB domain positioned at the N-terminus, accompanied by a variable number of zinc fingers arranged at the C-terminus.

Zinc finger with KRAB and SCAN domain 3 (ZKSCAN3/ZNF306) is a zinc finger transcription factor characterized by the presence of Krüppel-associated box (KRAB) and SCAN domains [17]. This gene, located on chromosome 6 at position 6p22.1, exhibits three distinct variants. Variant 1, which represents the longest transcript, produces the longer isoform (isoform 1). Both variant 1 and variant 2 encode identical proteins; however, variant 3 differs in its 5′ untranslated region (5′UTR), lacking a segment of the upstream coding region and initiating translation at a downstream start codon. As a result, variant 3 encodes a unique N-terminus and yields a shorter isoform protein (isoform 2) compared to isoform 1 (Figure 1) [17]. ZKSCAN3 plays a pivotal role in promoting tumor growth by modulating various cancer-related characteristics, including enhancing tumor cell proliferation, inhibiting apoptosis, facilitating invasion and metastasis, as well as suppressing autophagy in tumor cells.

This review provides a comprehensive summary of the current understanding of ZKSCAN3’s expression levels, functions, and the associated molecular mechanisms. We emphasize the clinical significance of ZKSCAN3, highlighting its potential as a biomarker for predicting cancer outcomes and its promise as an innovative therapeutic strategy for cancer treatment.

## 2. The Discovery History of ZKSCAN3

**Figure 2:**
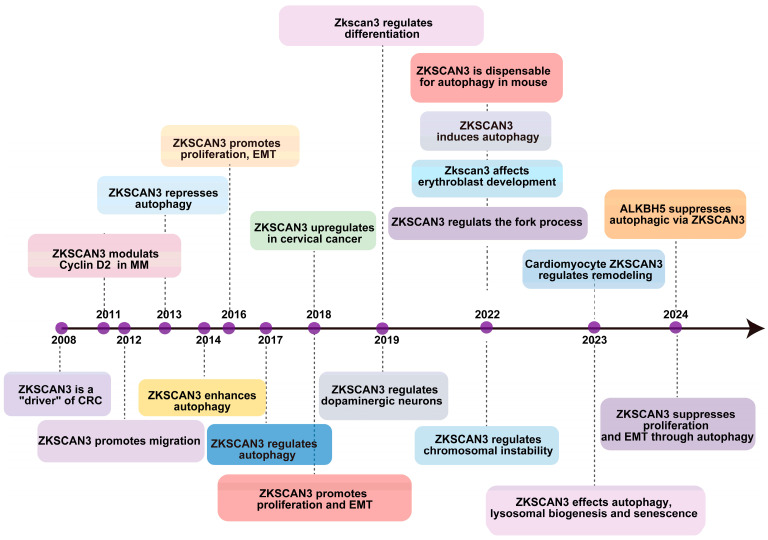
The timeline outlines key regulatory and biological discoveries pertaining to ZKSCAN3, presenting the major discoveries regarding its regulatory and physiopathological roles since 2008.

The initial identification of ZKSCAN3’s role in tumorigenesis was conducted by Yang et al. in 2008 [21], revealing its excessive expression in colorectal cancer (CRC). Their findings demonstrated that overexpressing this gene in tumor cells significantly enhanced their proliferation, both in vitro and through an orthotopic tumor model in vivo [21]. Furthermore, the overabundance of ZKSCAN3 correlates with elevated levels of several factors that contribute to colon cancer progression, including integrin, cyclin D1 (CCND1), epidermal growth factor receptor (EGFR), and vascular endothelial growth factor (VEGF) [22]. In 2013, Chauhan and colleagues established that the growth deficiency resulting from *ZKSCAN3* silencing through the combination of CRISPR/Cas9-mediated gene knockout is partially attributed to an increase in autophagy, ultimately leading to cellular senescence [23]. They also demonstrated that ZKSCAN3 directly impacts the transcriptional regulation of over 60 genes encoding proteins crucial for various stages of autophagy and lysosome formation and function [23]. As a newly discovered transcription factor, ZKSCAN3 enhances the production of proteins involved in diverse cellular processes, such as migration, invasion, apoptosis, proliferation, nucleolar maintenance, neoplastic transformation, cell differentiation, and autophagy. Its association with the progression of malignancies like bladder, breast, cervix, stomach, multiple myeloma, colon, prostate, and liver cancers has been established [24], suggesting its potential as a valuable prognostic marker in various cancer types. Recently, Li et al. discovered that ZKSCAN3 inhibits the transcription of microtubule-associated protein light chain 3-II (LC3-II), which is an autophagy-related gene [17], thus impeding autophagy’s various stages in different pathologies. Their investigation into acute pancreatitis (AP) revealed ZKSCAN3’s involvement in autophagy. Furthermore, it was identified that ALKBH5 exacerbates the severity of AP by mediating the inhibition of autophagic flux, which occurs through the demethylation of m6A in ZKSCAN3 mRNA [25], as illustrated in Figure 2.

## 3. The Reasons for the Dysregulation of ZKSCAN3

ZKSCAN3 dysregulation is a multifaceted phenomenon influenced by various mechanisms at both genetic and epigenetic levels, as well as through transcriptional regulation, post-translational modifications, and environmental factors. Specifically, genetic mutations—including point mutations, deletions, and copy number variations—can significantly affect protein structure, function, and expression levels [26]. Epigenetic modifications, such as DNA methylation and histone alterations, can lead to gene silencing by shRNA or activation, thereby resulting in the dysregulated expression of ZKSCAN3 [27]. Transcriptional regulation involves changes in transcription factors and regulatory non-coding RNAs like microRNAs that can modulate ZKSCAN3 expression [23,28,29]. Post-translational modifications—including phosphorylation, acetylation, and ubiquitination—can substantially influence protein stability and activity; these processes contribute to the dysregulation of ZKSCAN3 [30,31]. Furthermore, alterations in cellular signaling pathways such as the PI3K/AKT/mTOR and MAPK/ERK pathways can impact both the expression and functionality of ZKSCAN3 [32,33]. Environmental factors—including exposure to toxins, chemicals, radiation; inflammatory conditions; and chronic diseases—may also affect ZKSCAN3 expression via diverse signaling pathways [34,35,36]. Within the tumor microenvironment (TME), changes such as hypoxia and nutrient deprivation impose selective pressures on cancer cells that drive adaptation mechanisms potentially contributing to ZKSCAN3 dysregulation [23,33,35]. Immune responses within the TME further modulate ZKSCAN3 expression through cytokine signaling interactions with immune cells [34].

Moreover, ZKSCAN3 expressed in stromal cells within the TME may be particularly vulnerable to dysregulation due to paracrine signaling from cancer cells. This process involves the secretion of cytokines, growth factors, and extracellular vesicles that influence surrounding cellular environments. Changes in the tumor microenvironment, such as alterations in the composition and stiffness of the extracellular matrix, can significantly influence stromal cell function and ZKSCAN3 expression [28]. Chronic inflammation within this microenvironment may further modulate gene expression in stromal cells through inflammatory mediators [33], while hypoxia can impact gene expression via hypoxia-inducible factors [28]. Additionally, epigenetic modifications—including DNA methylation, histone modifications, and non-coding RNA-mediated regulation—can contribute to the dysregulated expression of ZKSCAN3 in stromal cells [23,28,29,33].

In summary, the dysregulation of ZKSCAN3 is a complex process that encompasses various mechanisms at genetic, epigenetic, transcriptional, and post-translational levels. Environmental factors play a crucial role as well, particularly within the tumor microenvironment, where stromal cells are affected by paracrine signaling, altered conditions of their surroundings, inflammatory responses, hypoxic states, and epigenetic changes.

## 4. The ZKSCAN3 Pathway in Different Types of Human Cancers

ZKSCAN3 expression is elevated in various organ cancers, including colon, prostate, bladder, breast, cervix, stomach, myeloma, and liver cancers, and is frequently associated with unfavorable prognostic outcomes [18,21,22,37,38,39,40,41,42]. Notably, ZKSCAN3 exhibits oncogenic properties across different cancer models. For instance, in the context of colorectal cancer (CRC), overexpression of *ZKSCAN3* has been shown to promote anchorage-independent growth in vitro, as well as tumor growth at the primary site and liver metastasis in vivo through an orthotopic tumor model [21]. Furthermore, it confers resistance to 5-fluorouracil treatment. A distinct study on CRC unveiled an association between the expression of ZKSCAN3 and the concentrations of cancer-related proteins, notably carcinoembryonic antigen (CEA), VEGF, and AKT, within liver metastases [43]. Interestingly, CEA further potentiates the invasive characteristics of *ZKSCAN3*-overexpressing cells [43].

In breast, bladder, and prostate cancer models, ZKSCAN3 has demonstrated an enhancing effect on cancer cell vitality, migration, invasiveness, and tumorigenicity [10,32,40]. While the precise molecular mechanisms underlying ZKSCAN3’s role in cancer remain elusive, its upregulated expression is linked to multiple downstream signaling pathways. For instance, ZKSCAN3 consistently promotes the expression of metalloproteinases 2 (MMP2) and 9 (MMP9). Specifically, in breast cancer, ZKSCAN3 expression exhibits a positive correlation with CCND1 and BCL2 expression, while it negatively correlates with the expression of Bcl2 Associated X Protein (Bax). Furthermore, inhibition of ZKSCAN3 has been found to hinder the phosphorylation and subsequent activation of the AKT/mTOR pathway [32]. Furthermore, in hepatocellular carcinoma (HCC), ZKSCAN3 facilitates epithelial-mesenchymal transition (EMT), as evidenced by changes in the expression patterns of EMT markers, including decreased E-cadherin and increased N-cadherin and vimentin levels. Additionally, ZKSCAN3 enhances cell motility and invasion in vitro, as well as metastasis in vivo, through the establishment of a pulmonary metastatic model. Mechanistically, ZKSCAN3 interacts with the promoter region of Integrin β4 (ITGβ4), thereby stimulating its production [24,44]. Integrin β4 (ITGβ4) subsequently triggers AKT phosphorylation through the non-receptor tyrosine kinase FAK, a crucial step for the efficient induction of epithelial-mesenchymal transition (EMT) [24]. Moreover, ZKSCAN3 engages with the promoter region of cyclin D2 (CCND2) in multiple myeloma, leading to its upregulated expression compared to normal samples [13]. In prostate cancer, ZKSCAN3 regulates cell cycle progression, attachment, migration, and motility. Moreover, ZKSCAN3 modulates cell proliferation, apoptosis, and tumorigenicity in several cancer types. These findings indicate that ZKSCAN3 regulates numerous molecules crucial for tumor development and progression, including CCND1, CCND2, EGF, IGF-2, integrin-β4, MMP2, MMP9, NF-κB, and VEGF [22].

**Figure 3:**
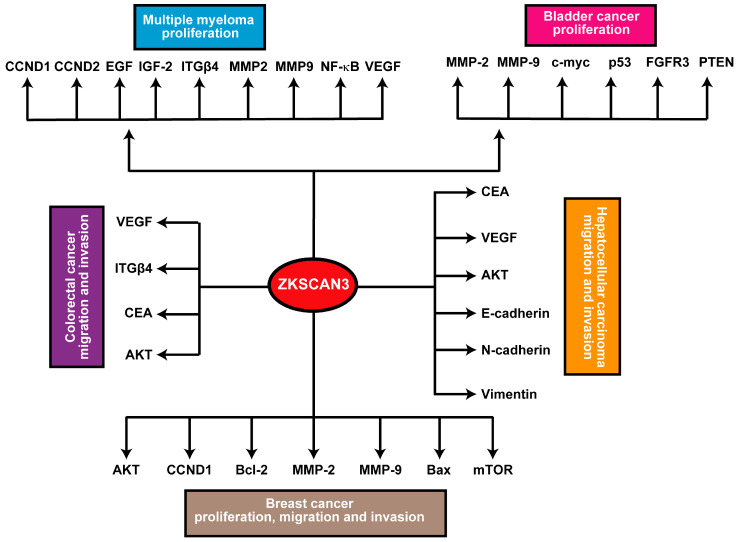
Schematic summary of the ZKSCAN3 pathway: Elevated expression levels of ZKSCAN3 significantly enhance cellular proliferation, migration, and invasive capabilities, ultimately contributing to the development and progression of these malignancies.

The BRAF inhibitor (BRAFi) hinders TFEB phosphorylation, thereby facilitating its nuclear translocation [35]. This inhibitory effect on TFEB phosphorylation is further enhanced by the elevated phosphorylation and subsequent deactivation of the ZKSCAN3 transcriptional repressor, mediated by JNK2 and p38-MAPK [45]. Separately, protein kinase C (PKC) activates the TFEB transcription factor while concurrently deactivating the ZKSCAN3 transcriptional repressor through distinct signaling cascades [35]. Activation of PKC results in the deactivation of GSK3β, leading to a reduction in TFEB phosphorylation, promoting its nuclear translocation and activation. Furthermore, PKC stimulates JNK and p38 MAPK, which phosphorylate ZKSCAN3, causing its inactivation and relocation out of the nucleus [46]. Finally, recombinant proteins belonging to the Wnt gene family or the GSK inhibitor CHIR 99021 upregulate ZKSCAN3 expression in a β-catenin-dependent manner through activation of the Wnt pathway [41], as depicted in Figure 3.

## 5. Expression and Regulation of ZKSCAN3 in Different Types of Human Cancers

ZKSCAN3 plays a pivotal function in modulating gene expression pertinent to diverse cellular processes, such as proliferation, apoptosis, metabolism, and differentiation [23,32,41]. Recent studies have implicated ZKSCAN3 in oncogenesis, highlighting its potential utility as both a biomarker and a therapeutic target in cancer. Nevertheless, the expression patterns and regulatory mechanisms of ZKSCAN3 across different human cancer types remain to be clarified.

### 5.1. Expression of ZKSCAN3 in Different Types of Human Cancers

When compared to adjacent normal tissues, ZKSCAN3 was found to be markedly overexpressed in several solid tumors, including colorectal cancer, gastric cancer, pancreatic ductal adenocarcinoma (PDAC), and non-small cell lung cancer (NSCLC). Conversely, hematological cancers, such as leukemia and lymphoma, exhibited variable expression levels of ZKSCAN3, with certain subtypes demonstrating downregulation [25,47,48]. Elevated ZKSCAN3 expression was associated with both poorer overall survival and disease-free survival among patients with colorectal and gastric cancer, indicating its potential utility as a prognostic marker [38]. Comprehending the regulatory mechanisms governing ZKSCAN3 expression is of crucial importance for deciphering its role in cancer.

#### 5.1.1. Transcriptional Regulation

Numerous reports have emerged concerning the transcriptional regulation of ZKSCAN3, primarily encompassing the activation of promoters and epigenetic modifications. Among these, the regulation of promoter activation by ZKSCAN3 is delineated as follows: The promoter region of the ZKSCAN3 gene is furnished with specific binding sites for pivotal oncogenic transcription factors, notably c-Myc and NF-κB, both of which are commonly upregulated in a vast array of cancers. The binding of c-Myc, a master regulator of cellular proliferation and metabolism, and NF-kB, a key element in inflammatory responses and cell survival, to the ZKSCAN3 promoter augments the transcriptional activation of ZKSCAN3. This regulatory interaction suggests that ZKSCAN3 functions as a downstream mediator in the oncogenic pathways orchestrated by these transcription factors, thereby facilitating tumor growth, survival, and metastasis. The upregulation of ZKSCAN3 in response to c-Myc and NF-kB not only emphasizes its role in facilitating aggressive cancer phenotypes but also highlights its potential as a therapeutic target [49,50]. The regulation of epigenetic modifications by ZKSCAN3 is stated as follows: The promoter region of the ZKSCAN3 gene demonstrates high levels of methylation in certain cancer types, highlighting a sophisticated regulatory landscape that encompasses both activation and repression mechanisms. DNA methylation, a common epigenetic modification, typically functions to suppress gene expression by impeding the binding of transcription factors and recruiting repressive chromatin remodelers. In the context of ZKSCAN3, hypermethylation of its promoter in specific cancers implies a potential mechanism for downregulating its expression, which might be necessary to modulate its oncogenic functions under certain conditions. However, the presence of high methylation, along with evidence of *ZKSCAN3* overexpression in other cancer contexts, indicates that the regulatory control of ZKSCAN3 is multifaceted. This dual regulation could involve interaction with other epigenetic modifications, transcriptional activators, or microenvironmental factors that override the repressive effects of methylation. Such complexity emphasizes the necessity of a nuanced understanding of ZKSCAN3 regulation, as it may vary significantly between different cancer types and stages [17,27,29,51]. By elucidating the equilibrium between methylation-mediated repression and other activating signals, researchers can better comprehend how ZKSCAN3 contributes to tumorigenesis and identify potential therapeutic targets that disrupt these complex regulatory networks.

#### 5.1.2. Post-Transcriptional Regulation

The post-transcriptional regulation of ZKSCAN3 is predominantly linked to the modulation of microRNAs (miRNAs) and alternative splicing. Specifically, the regulation of miRNAs encompasses numerous specific miRNAs. The promoter region of the ZKSCAN3 gene is subject to elaborate post-transcriptional regulation mediated by several microRNAs (miRNAs), notably including miR-124. These miRNAs bind to complementary sequences within the 3′ untranslated region (3′ UTR) of ZKSCAN3 mRNA, facilitating either the degradation of the mRNA or the inhibition of its translation, thereby proficiently downregulating ZKSCAN3 expression in specific cellular contexts. miR-124 is widely recognized for its role in suppressing epithelial-mesenchymal transition (EMT) and maintaining epithelial integrity, and its targeting of ZKSCAN3 implies a mechanism by which it can inhibit tumor progression and metastasis by reducing ZKSCAN3-mediated oncogenic activities. Similarly, miR-34a, a crucial element in the p53 tumor suppressor network, contributes to the inhibition of cell proliferation and induction of apoptosis by downregulating ZKSCAN3, thereby hindering cancer cell survival and growth [21,49]. The downregulation of ZKSCAN3 by these miRNAs occurs predominantly in specific cancer types and stages, highlighting a context-dependent regulatory framework that maintains the balance of ZKSCAN3’s oncogenic functions. This miRNA-mediated control of ZKSCAN3 not only emphasizes the complexity of gene regulation in cancer but also presents potential therapeutic approaches where restoring or mimicking the activity of these miRNAs could suppress ZKSCAN3 expression and alleviate its contribution to tumorigenesis [24,30]. Furthermore, investigations have disclosed the presence of multiple alternative splicing isoforms of ZKSCAN3, which may exert disparate functional implications in cancer cells. The ZKSCAN3 gene undergoes alternative splicing, giving rise to multiple transcript variants that encode distinct protein isoforms, each potentially possessing distinctive functional attributes within cancer cells [21]. These alternative splicing events can give rise to variations in the protein domains, such as modifications in the zinc finger regions or the KRAB and SCAN domains, which might influence ZKSCAN3’s capacity to bind DNA, interact with other transcriptional regulators, and modulate gene expression in diverse manners [28]. In the context of cancer, these splicing variants could contribute to the heterogeneity of tumor behavior by influencing processes such as cell proliferation, apoptosis, metastasis, and metabolic reprogramming in an isoform-specific manner. For instance, certain splice variants might potentiate oncogenic pathways more effectively or evade regulatory mechanisms that suppress tumor growth, thereby facilitating more aggressive cancer phenotypes. Additionally, the existence of distinct ZKSCAN3 isoforms could affect the responsiveness of cancer cells to targeted therapies, as some variants may confer resistance or sensitivity to specific treatments.

#### 5.1.3. Post-Translational Modifications

The post-translational modifications governed by ZKSCAN3 predominantly encompass the regulation of phosphorylation and ubiquitination. ZKSCAN3 undergoes phosphorylation and ubiquitination, which affects its stability and nuclear localization, thereby modulating its transcriptional activity. Phosphorylation of ZKSCAN3 is typically mediated by specific kinases that add phosphate groups to serine, threonine, or tyrosine residues, resulting in conformational changes that can either enhance or inhibit its capacity to bind DNA and interact with other transcriptional co-regulators. This modification can also exert an influence on the nuclear import and export of ZKSCAN3, ensuring its presence in the nucleus, where it fulfills its function as a transcription factor. Concurrently, ubiquitination of ZKSCAN3 involves the attachment of ubiquitin molecules by E3 ubiquitin ligases, marking the protein for proteasomal degradation. This process not only governs the intracellular levels of ZKSCAN3, preventing excessive transcriptional activation of its target genes but also has an impact on its nuclear retention and turnover [17,29,52,53,54]. The equilibrium between phosphorylation and ubiquitination is of vital significance for maintaining the appropriate activity of ZKSCAN3; the dysregulation of these modifications may give rise to either the accumulation of ZKSCAN3, promoting oncogenic gene expression programs or its premature degradation, potentially disrupting normal cellular functions. In the context of cancer, aberrant phosphorylation and ubiquitination of ZKSCAN3 can lead to altered transcriptional landscapes that facilitate tumor growth, survival, and metastasis by modulating genes implicated in cell cycle progression, apoptosis inhibition, and metabolic reprogramming. Comprehending the precise mechanisms and regulatory networks governing ZKSCAN3’s phosphorylation and ubiquitination not only clarifies its role in cancer biology but also identifies potential therapeutic targets for disrupting its oncogenic functions through the modulation of these post-translational modifications.

### 5.2. Regulation of ZKSCAN3 in Different Types of Human Cancers

ZKSCAN3 has emerged as a pivotal factor in promoting the growth of colon cancer cells, both in vitro and in vivo, through an orthotopic tumor model. Its possession of tandem zinc fingers, a common trait in transcription factors, suggests involvement in regulating gene expression for tumor progression. Utilizing a nucleotide library, the DNA recognition motif of ZKSCAN3 was identified as KRDGGG [44]. Expression profiling revealed an upregulation of 204 genes (ranging from two to twenty-nine folds) and a downregulation of 76 genes (by two to five folds). Among the upregulated targets were genes related to growth, such as MSTlR, MEK2, RasGRP2, IGF2, ITGβ4, VEGF, MMP2, cathepsin D, and PRSS3 [44]. In human malignancies, such as colon and prostate cancer, ZKSCAN3 functions as an oncogenic transcription factor.

ZKSCAN3 facilitates epithelial-mesenchymal transition (EMT), evident from the distinct EMT marker expression, enhanced cellular motility and invasion in vitro, and intensified metastasis in vivo through the establishment of a pulmonary metastatic model. Mechanistically, ZKSCAN3 binds to the ITGβ4 promoter, stimulating its expression. Through the action of FAK, a non-receptor tyrosine kinase, ITGβ4 subsequently stimulates the phosphorylation of AKT [24]. This signaling cascade is crucial for EMT induction [13]. ZKSCAN3 expression modulates ITGβ4 levels, binding to its promoter both in vitro and in vivo through the establishment of a pulmonary metastatic model. Knockdown of ITGβ4 using shRNA counteracted ZKSCAN3-induced anchorage-independent colony formation [44]. Additionally, ZKSCAN3 augments the FAK/AKT pathway through direct binding to the ITGβ4 promoter, while miR-124 regulates ZKSCAN3 expression in HCC cells [22].

ZKSCAN3 interacts with the *CCND2* and *VEGF* promoters, activating their expression in multiple myeloma. Expression levels of ZKSCAN3 are significantly higher in myeloma samples compared to normals [13,44]. *ZKSCAN3* overexpression stimulates *CCND2* promoter activity independently of MAF/MAFB, regulated by the number of ZKSCAN3 binding sites [22]. ZKSCAN3 expression exhibits a correlation with CCND2 levels in both cell lines and primary samples, stimulating CCND2 production. ZKSCAN3 selectively binds to *CCND2* promoter sequences in myeloma cells [18]. Furthermore, ZKSCAN3 represses the spindle checkpoint protein MAD2L2, and disruption of ZKSCAN3 may lead to chromosomal instability (CIN) via MAD2L2-dependent pathways [41]. During replication stress, ZKSCAN3 serves to protect replication forks and maintain chromosomal stability. The absence of ZKSCAN3 leads to excessive removal of forks by Exo1 and Mre11, notably in BRCA1-deficient cells. ZKSCAN3 fulfills dual roles in fork resection, which are mediated by its SCAN box domain, whereas its Krüppel-associated and zinc finger domains are not essential for these functions. Furthermore, the loss of ZKSCAN3 does not impact the short-term survival of either BRCA1-proficient or -deficient cells when treated with PARPi, cisplatin, or hydroxyurea [55].

ZKSCAN3 regulates autophagy and lysosome biogenesis, controlling around 60 genes involved in these processes [23]. Autophagy, a universal process involving lysosomal degradation, is now recognized as a complex system involving gene expression and histone modifications [30,56]. ZKSCAN3 suppression activates autophagy and lysosome formation, affecting genes like ULK1, Map1lC3b, DFCP, and WIPI2. ZKSCAN3 and TFEB exhibit opposing effects on autophagy and lysosome biogenesis, indicating collaborative action [35]. The oncogenic protein CHD1L promotes HCC migration and metastasis via autophagy, inhibiting ZKSCAN3 transcription. CHD1L-induced migration and metastasis are facilitated by Paxillin degradation, promoted by ZKSCAN3 [18]. Adhikari et al. investigated a study to explore the synergistic impact of cold atmospheric plasma (CAP) and silymarin nanoemulsion (SN) on G-361 melanoma cells. Their findings revealed that this combination induced autophagy and modulated autophagy-related transcriptional factors and genes, notably ZKSCAN3 [56].

**Figure 4:**
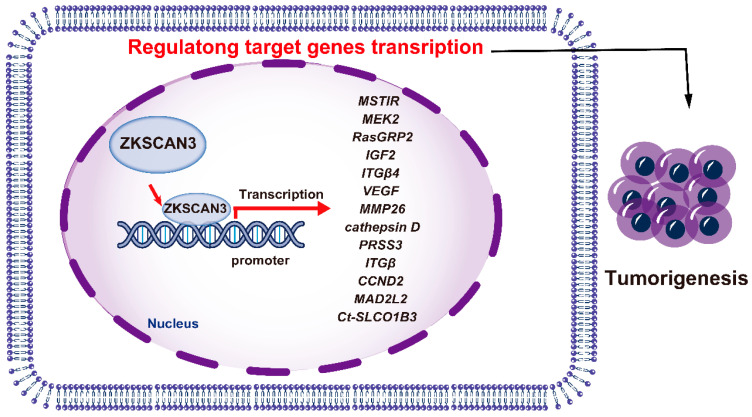
The mechanisms of ZKSCAN3 promoting cancer progression.

ZKSCAN3 also exerts a pivotal function in modulating the expression of the solute carrier organic anion transporter family member 1B3 (SLCO1B3) gene in particular cell types. Notably, potential binding sites for ZKSCAN3 have been detected within a 100-base pair region upstream of the transcriptional start site, located in the core promoter region of Ct-SLCO1B3 fragments [57]. Introduction of mutations into the ZKSCAN3 binding region resulted in a decrease in luciferase activity for the Ct-SLCO1B3 reporter gene constructs in the CRC cell lines DLD1 and T84, reducing activity to 29.9% and 14.3%, respectively. However, when liver-derived Hep3B cells were utilized, a residual activity of 71.6% was observed [57]. ZKSCAN3, therefore, modulates the expression of multiple genes that facilitate tumor progression (Figure 4).

## 6. Biological Roles of ZKSCAN3 Regulates Various Hallmarks of Cancer

**Figure 5:**
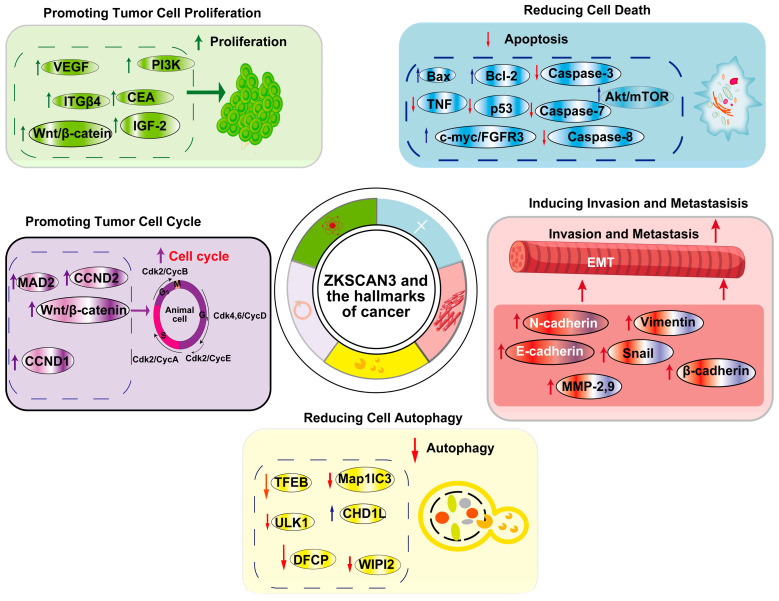
ZKSCAN3 and the Hallmarks of Cancer: This article primarily focuses on five cancer hallmarks regulated by ZKSCAN3, namely promoting tumor cell proliferation, decreasing cell death, enhancing the tumor cell cycle, reducing cell autophagy, and inducing invasion and metastasis.

ZKSCAN3 (ZNF306) is a zinc finger transcription factor that plays a pivotal role in cancer progression by modulating multiple hallmarks of the disease. It acts as a transcriptional regulator, influencing genes involved in cell proliferation, apoptosis, autophagy, angiogenesis, invasion, and metastasis (Figure 5). Overexpression of *ZKSCAN3* has been observed across various cancers, where it promotes tumor growth by enhancing cellular proliferation and inhibiting programmed cell death mechanisms (Table 1). Notably, ZKSCAN3 represses autophagy-related genes, enabling cancer cells to survive under metabolic stress by preventing the degradation of essential cellular components. Additionally, it contributes to angiogenesis by upregulating pro-angiogenic factors that facilitate an increased blood supply to the tumor microenvironment [24]. Furthermore, ZKSCAN3 enhances invasion and metastasis through the regulation of genes associated with extracellular matrix remodeling and cell adhesion [24,44]. This enables cancer cells to disseminate to distant organs effectively. Through these mechanisms, ZKSCAN3 orchestrates a network of signaling pathways that collectively support tumor development and progression, underscoring its potential as a therapeutic target in cancer treatment and further emphasizing its multifaceted role in cancer biology.

### 6.1. ZKSCAN3 Promotes Tumor Cell Proliferation

ZKSCAN3 serves as a crucial factor in driving the proliferation of tumor cells, a hallmark of tumorigenesis that is often characterized by deregulated cell growth [60,61]. Elevated expression levels of ZKSCAN3 have been documented across multiple cancer types, particularly in association with the accelerated growth of tumor cells.

Within the scope of colorectal cancer (CRC), ZKSCAN3 demonstrates heightened expression in invasive cells and modulates the expression of genes, including VEGF and ITGβ4, which contribute to tumor growth [44]. Notably, the correlation between ZKSCAN3 and CRC liver metastasis (CLM) indicates its involvement in invasive signaling pathways. The overexpression of *ZKSCAN3* correlates favorably with the expression of CEA, VEGF, and AKT [43], enhancing the invasiveness of HCT116 cells overexpressing *ZKSCAN3* when exposed to CEA-coated filters compared to phosphate buffered saline (PBS)-treated controls. Additionally, the expression of ZKSCAN3 protein is elevated in hepatic metastatic tissue compared to primary tumor tissue in patients with high serum CEA levels, while it remains stable or decreases in those with normal levels. This suggests that ZKSCAN3 facilitates the progression and infiltration of colorectal tumors, particularly in the presence of CEA-producing tumors [43]. As a result, ZKSCAN3 represents a crucial factor in CRC progression and holds potential as a diagnostic and therapeutic target.

The cell cycle, a vital mechanism governing cell reproduction [62,63,64], is significantly influenced by ZKSCAN3. This protein possesses the capacity to arrest cell division, inducing cells to enter the G0/G1 resting phase [22]. This arrest reduces cell growth and ultimately triggers cell death. Notably, suppressing *ZKSCAN3* halts cancer cell progression through the G0/G1 phase by inhibiting the Wnt/β-catenin signaling pathway [65,66]. Furthermore, ZKSCAN3 functions as a transcriptional regulator of MAD2L2, a mitotic spindle checkpoint protein. Overexpression of *ZKSCAN3* reduces MAD2L2 production by decreasing its promoter activity [22]. This results in the onset of chromosomal instability (CIN) in colon cancer cells. On the other hand, the deletion or inhibition of ZKSCAN3 enhances MAD2L2 expression and retards cell cycle progression [41].

ZKSCAN3 also modulates critical pathways involved in cell proliferation, including CCND1, CCND2, EGF, IGF-2, ITGβ4, MMP2, MMP9, NF-κB, and VEGF. In prostate cancer cell lines, *ZKSCAN3*-shRNA significantly reduces colony formation, cell viability, invasion, migration, and the expression of MMP2 and MMP9 while inducing apoptosis [40]. Furthermore, ZKSCAN3 expression exhibits a strong correlation with aggressive histopathological characteristics and an elevated risk of tumor recurrence, functioning as an independent predictive marker [37].

### 6.2. ZKSCAN3 and Its Role in Cell Death

ZKSCAN3 initiates cell death through a highly regulated process known as apoptosis, which is orchestrated by a cascade of signaling events aimed at preserving cellular homeostasis [67,68,69]. Apoptosis plays a pivotal role in clinical cancer therapy, particularly in the elimination of cancer cells [70,71]. This process can be broadly categorized into two mechanisms: the intrinsic and extrinsic apoptotic pathways [72]. The intrinsic pathway, also termed mitochondria-mediated apoptosis, involves the interaction of pro-apoptotic B-cell lymphoma 2 (Bcl-2) proteins, anti-apoptotic Bcl-2 proteins, and BH3-only proteins, culminating in the activation of executioner caspases 3 and 7 through caspase 8 stimulation [73]. Conversely, the extrinsic pathway is regulated by death receptors, including the tumor necrosis factor (TNF) receptor, which initiates the activation of caspase 9, subsequently leading to the activation of executioner caspases [61,74].

Previous studies have demonstrated that ZKSCAN3 potentiates intrinsic apoptosis through multiple pathways. The inhibition of *ZKSCAN3* using shRNA in MCF-7 and MDA-MB-231 cells diminishes cell viability, migration, and invasion, highlighting that *ZKSCAN3*-shRNA significantly decreases the expression of MMP-2, MMP-9, CCND1, and Bcl-2 while augmenting the expression of Bax in breast cancer cells [22]. Furthermore, the expression of *ZKSCAN3*-shRNA profoundly inhibits tumor growth in mice harboring breast cancer xenografts, indicating that suppressing ZKSCAN3 effectively impedes the Akt/mTOR signaling pathway by suppressing the expression of p-Akt and p-mTOR proteins in breast cancer cells. These findings underscore the substantial impact of ZKSCAN3 on breast cancer progression [32].

Suppressing *ZKSCAN3* in UMUC3 and 647V bladder carcinoma cells induces apoptosis by significantly reducing colony formation, cell viability, invasion, and migration. This also leads to a decrease in the expression of oncogenes c-myc/FGFR3, MMP-9, and MMP-2, coupled with an increase in apoptosis and the expression of tumor suppressor genes p53/PTEN [49]. Additionally, *ZKSCAN3*-shRNA expression profoundly inhibits the initiation and progression of tumors in mice bearing xenografts, highlighting the significant impact of ZKSCAN3 on bladder cancer growth and indicating that inhibiting ZKSCAN3 could serve as a viable therapeutic approach for treating bladder cancer [41,49]. Beyond its role in intrinsic apoptosis, ZKSCAN3 also possesses the ability to activate the extrinsic apoptosis pathway, as evidenced by its activation of the Bcl2 signaling pathway and induction of apoptosis in bladder and breast cancer [32,40].

Autophagy is a cellular process where cells undergo self-destruction by transferring proteins or organelles from the cytoplasm to the lysosome [71,75,76]. This process serves to fulfill the metabolic and survival requirements of both the organelles and the cell as a whole [75,77]. Prior research has shown that ZKSCAN3 can reduce autophagy by inhibiting the expression of autophagy-related proteins, resulting in decreased levels of autophagy-related proteins such as ULK1, Map1lC3b, DFCP, and WIPI2 [17]. Therefore, further research is warranted to elucidate the effects of ZKSCAN3 regulation on autophagy.

### 6.3. ZKSCAN3 Positively Regulates Tumor Cells Invasion and Metastasis Potentials

ZKSCAN3 actively modulates the invasiveness and metastatic proclivity of tumor cells. Metastasis, defined as the dissemination of neoplastic cells from the primary tumor site to colonize and subsequently grow in distant body parts, is orchestrated by a complex network of biological events. Our understanding of the regulatory mechanisms underlying metastasis has significantly advanced [78,79,80]. Epithelial-to-mesenchymal transition (EMT), the initiating and pivotal event in cancer metastasis [81,82], involves epithelial cells acquiring mesenchymal features, such as reduced intercellular adhesion and enhanced motility while losing their epithelial identity [47,80,83]. ZKSCAN3 enhances the EMT and metastatic potential of colorectal cancer (CRC) cells via the ITGβ4/FAK/AKT pathway [24], leading to increased expression of E-cadherin, a marker of epithelial identity, and decreased expression of mesenchymal markers like N-cadherin and vimentin, as well as cell migration markers MMP-2 and MMP-9 [24,43]. Furthermore, ZKSCAN3 expression correlates with cancer-related proteins like CEA, VEGF, and AKT levels in liver metastasis, with CEA further augmenting the invasive nature of *ZKSCAN3*-overexpressing cells [43]. In breast cancer cells, ZKSCAN3 elevates migration and invasion abilities through the Akt/mTOR signaling pathway by suppressing p-Akt and p-mTOR expression, thereby promoting proliferation, migration, and invasion [23,24,84]. Additionally, in hepatocellular carcinoma (HCC), ZKSCAN3 stimulates ITGβ4 expression by binding to its promoter, enhancing AKT phosphorylation, and triggering a signaling cascade essential for EMT. Moreover, ZKSCAN3 facilitates EMT by activating non-receptor tyrosine kinase FAKs, leading to AKT phosphorylation [24].

Clinical data from CRC patients validate the association between ZKSCAN3 and metastasis, particularly liver metastasis, as elevated ZKSCAN3 levels correlate with this type of metastasis [24,43]. Specifically, hepatic metastatic tissue exhibits higher ZKSCAN3 protein expression compared to primary tumor tissue in patients with high serum CEA levels, while in patients with normal serum CEA, ZKSCAN3 expression either declines or remains comparable. Additionally, a study of 450 individuals with sporadic CRC revealed a correlation between ZKSCAN3 alleles and male gender, family history of malignancy, high blood CEA concentration, and stage IV CRC, suggesting that ZKSCAN3 contributes to the progression and infiltration of colorectal tumors, especially facilitating CRC metastasis to the liver, particularly in CEA-producing tumors [43].

## 7. ZKSCAN3 Is a Potential Biomarker for Tumor Prognosis

**Figure 6:**
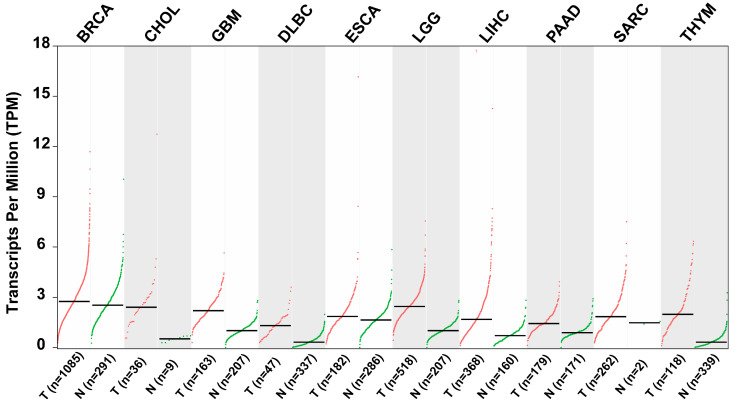
Scatter plots illustrating the relative expression levels of ZKSCAN3 in various tumor tissues compared to adjacent non-tumor tissues (data sourced from GEIPA: http://gepia.cancer-pku.cn/detail.php?gene=ZKSCAN3, accessed on 10 October 2024).

Elevated expression of ZKSCAN3 has consistently been associated with unfavorable clinical outcomes across various types of malignancies [38]. As depicted in Table 2 and Figure 6, scientific validation underscores ZKSCAN3’s anti-tumor properties and its potential as a clinically significant prognostic factor in diverse human cancers [32,38,39,40].

In cervical cancer patients, heightened expression of ZKSCAN3 is linked to reduced overall survival (OS) rates and a higher risk of tumor recurrence. Lee et al. further demonstrated significant upregulation of ZKSCAN3 in cervical cancer tissues compared to adjacent non-neoplastic cervical mucosa. Additionally, gene copy number analysis revealed an increased number of ZKSCAN3 copies in cervical cancer samples. Notably, in low-grade cervical carcinoma, elevated ZKSCAN3 expression was positively correlated with OS and progression-free survival compared to wild-type cases [39].

Takano et al. detected ZKSCAN3 in 32.2% of tumor samples, with a significant association observed between its presence and lymphatic invasion as well as the development of distant metastases. Patients with ZKSCAN3-positive tumors exhibited lower OS rates compared to those with ZKSCAN3-negative tumors. Multivariate analysis further confirmed ZKSCAN3 as an independent prognostic factor for OS [38]. Furthermore, ZKSCAN3 expression is directly associated with shorter OS, relapse-free survival, and an unfavorable prognosis in prostate cancer, gastric carcinoma, and HCC [32,38,39,40]. While previous studies have explored the role of ZKSCAN3 in various cancers, no study has investigated the sequential genetic alterations in myeloma genomes during the transition from diagnosis to plasma cell leukemia. Egan et al. identified five recently acquired single nucleotide variations (SNVs), including truncating mutations in RB1 and ZKSCAN3, in myeloma patients undergoing leukemic transformation. These findings provide insights into the leukemic transition events and the progression of high-risk myeloma patients over time using whole-genome sequencing. The study also revealed potential mutations contributing to the development of myeloma and its transformation into extramedullary diseases, such as secondary plasma cell leukemia (sPCL) [48]. Collectively, these findings establish a direct link between ZKSCAN3 and tumor progression and prognosis, indicating its potential as a biomarker for predicting malignant outcomes.

## 8. Conclusions and Perspectives

ZKSCAN3 emerges as a potential driver of tumor growth, capable of regulating diverse cancer characteristics, including cell proliferation, apoptosis, invasion, metastasis, and autophagy (Figure 5). Elevated expression of ZKSCAN3 is observed in numerous malignant tumors, such as glioma, HCC, colorectal cancer (CRC), and breast cancer. As depicted in Figure 4, ZKSCAN3 regulates tumor growth through transcriptional mechanisms. However, the precise role of ZKSCAN3 in processes like autophagy remains elusive due to inconsistent findings in current research, necessitating further in-depth investigations.

Recent studies have revealed that ZKSCAN3 promotes senescence in human mesenchymal stem cells (hMSCs) by maintaining heterochromatin stability through interactions with heterochromatin- and nuclear lamina-associated proteins [28]. Lysosomal biogenesis, a crucial adaptive process for cellular homeostasis, has been targeted as a therapeutic strategy for lysosomal-related disorders [85,86]. Ding et al. established a lysosome-based screening method to identify bioactive compounds that enhance lysosomal biogenesis, revealing Hdj-23, a triterpene, as an activator of TFEB/TFE3 and inducer of lysosome biogenesis [62]. Interestingly, ZKSCAN3 exhibits opposing effects in different cellular contexts. For instance, in cardiomyocytes, *ZKSCAN3* knockout disrupts the balance between autophagy activation and inhibition, leading to exacerbated cardiac remodeling following transverse aortic constriction [87]. Conversely, ZKSCAN3 and its Drosophila homolog M1BP share comparable roles in inhibiting autophagy, highlighting the evolutionary conservation of autophagy regulation through transcriptional control [29]. In pancreatic cancer, ZKSCAN3 expression correlates positively with overall survival, yet its suppression enhances tumor cell growth, migration, and invasion [54]. ZKSCAN3 also plays a role in bacterial infections, as evidenced by its upregulation in mice with Pseudomonas aeruginosa-induced pneumonia, resulting in impaired phagosome and lysosome function [88]. Notably, ZKSCAN3 has implications in neurodegenerative disorders like Parkinson’s disease (PD), where autophagy-lysosome dysregulation contributes to disease pathogenesis [89,90,91]. Wu et al. demonstrated that SIRT1-ZKSCAN3 pathway activation mitigated MPP (+)-induced cellular damage in PD models, suggesting a novel mechanism for controlling ZKSCAN3 nuclear translocation [34]. Genetic mutations in α-synuclein, a PD-associated protein, impede autophagy through ZKSCAN3 nuclear translocation, dependent on the JNK pathway [51,91,92,93,94,95,96,97]. In acute kidney injury, ZKSCAN3 negatively regulates autophagy activity in cancer tissues but not in non-tumor tissues [53,98,99,100,101], indicating context-specific roles. Vasectomy-associated prostate cancer risk may be linked to elevated ZKSCAN3 expression [102,103,104], while ZKSCAN3 regulates lysosomal function and autophagy in retinal pigment epithelial cells [33]. Pulmonary function assessments implicate ZKSCAN3 in respiratory health [105], further emphasizing its diverse biological functions. Soler Artigas et al. have found significant connections with pulmonary function near or within the genes, including ZKSCAN3 [36].

An increasing body of evidence indicates that ZKSCAN3 plays a pivotal role in tumour biology and may have a significant impact on clinical practice. It has been demonstrated that the expression levels of ZKSCAN3 in a diverse range of cancer types are closely correlated with a variety of clinical outcomes. This suggests that its presence may influence tumour progression, metastasis, and patient prognosis. In particular, in certain malignant tumours, high levels of ZKSCAN3 expression have been found to be associated with poor survival, which significantly demonstrates its potential as a prognostic indicator. Furthermore, ZKSCAN3 is implicated in a number of pivotal cellular processes, including cell proliferation, apoptosis, and epithelial-mesenchymal transition, underscoring its potential as a therapeutic target. It is anticipated that intervention against ZKSCAN3 in cancer therapy will provide novel therapeutic strategies, particularly in cases of cancer where aberrant ZKSCAN3 expression is present. As our understanding of the function and regulation of ZKSCAN3 deepens, its integration into prognostic models and therapeutic regimens could lead to improved patient management and the development of more personalised oncology treatment strategies.

Overall, ZKSCAN3’s novel roles in various diseases offer insights into the molecular mechanisms governing disease progression. Future research should explore ZKSCAN3’s involvement in additional cancer hallmarks and potential exceptions to its pro-tumorigenic role. Although the oncogenic function of ZKSCAN3 was initially identified in 2008, and its pivotal role in various tumorigenesis and its regulatory mechanism in tumor control has been reported, the role of ZKSCAN3 in molecular interactions has scarcely been reported. Given that research related to molecular interactions regarding ZKSCAN3 is still highly restricted and superficial, future studies on the molecular interactions of ZKSCAN3 merit further exploration. Nonetheless, current findings suggest that ZKSCAN3 holds promise as a diagnostic biomarker and anti-tumor therapeutic target.

## Figures and Tables

**Table 1:** Biological implications of ZKSCAN3 on cancer hallmarks.

Tumor	Cell Lines	Hallmarks of Cancer	Phenotypes	Mechanisms	Refs
BLCA	UC13, UMUC3, 647V, 5637	Sustained proliferative signaling, senescence	ZKSCAN3 regulated autophagy, cell proliferation, migration, invasion	ZKSCAN3-shRNA promotes membrane blebbing, represses cell growth, and induces senescence and autophagy by MMP-2, MMP-9, c-myc/FGFR3, p53/PTEN	[23,49]
CESC	HeLa, C33a, Caski, HeLa, SiHa	Sustained proliferative signaling	ZKSCAN3 enhanced proliferation	ZKSCAN3 cannot promote the expression of autophagic and lysosomal genes	[14,24]
CRC	HCT116, LoVo, LS174T, SW480, RKO, CT26, MCA38	Sustained proliferative signaling, metastasis	ZKSCAN3 enhanced proliferation, invasion, and metastasis regulate erythroblast development.	ZKSCAN3 negatively regulated the formation of plasma cells by modulating the transcription of GATA1; ZKSCAN3 promotes anchorage-independent growth and orthotopic tumor growth; VEGF and ITGβ4, CEA, and AKT	[21,41,43,44,58,59]
MM	RPMI 8226	Sustained proliferative signaling	ZKSCAN3 enhanced proliferation	ZKSCAN3 modulated CCND2 expression in MM	[22,56]
BRCA	MCF-7 and MDA-MB-231	Sustained proliferative signaling	ZKSCAN3 regulated cell viability, migration, invasion	ZKSCAN3 influenced the expression of CCND1, Bcl-2, MMP-2, MMP-9, Bax, and Akt/mTOR signaling pathways	[32]
PCA	PC3, LNCaP, VcaP, PC3, DU145, C4-s2	Sustained proliferative signaling, metastasis	ZKSCAN3 promoted PCA migration	None	[37,40]
GBM	LN18	Sustained proliferative signaling	ZKSCAN3 promoted GBM autophagy	NOP53 suppresses autophagy through ZKSCAN3-dependent and -independent pathways	[52]

**Abbreviations:** BLCA: bladder carcinoma; CESC: cervical Cancer; CRC: colorectal cancer; MM: multiple myeloma; BRCA: breast carcinoma, PCA: Prostate cancer; GBM: glioblastoma multiforme; CCND1: Cyclin D1; CCND2: Cyclin D2; ITGβ4: Integrinβ4; Bcl-2: B-cell lymphoma 2; Bax: Bcl2 Associated X Protein.

**Table 2:** ZKSCAN3 expression and relevant clinical characteristics in human cancer.

Cancer Type	Expression	Relevant Clinical Characteristics	Refs
Gastric	Upregulated	Worse OS, poorer clinical prognosis	[38]
HCC	Upregulated	Shorter OS and DFS in HCC patients	[24]
Colorectal	Upregulated	pathogenesis	[21,41]
Myeloma	Upregulated	pathogenesis	[22]
Breast cancer	Upregulated	ZKSCAN3 is significantly associated with lymph node metastasis, differentiation, increased tumor size, and deterioration of TNM staging	[32]
Bladder cancer	Upregulated	pathogenesis	[49]
Uterine Cervical Cancer	Upregulated	*ZKSCAN3* overexpression was also significantly associated with poor OS of the patients	[39]
Prostate Cancer	Upregulated	ZKSCAN3 enhances the migration of prostate cancer cells, and ZKSCAN33 tumor patients have a significantly higher risk of biochemical recurrence after radical prostatectomy.	[37,40]

**Abbreviations**: OS: Overall survival; TNM: Tumor-nodes-metastasis; DFS: Disease-free survival.

## Data Availability

Not applicable.

## Abbreviations

abridgement	generic term
ZNFs	Zinc finger proteins
ZKSCAN3	Zinc finger with KRAB and SCAN domain 3
KRAB	Krüppel-associated box
CRC	colorectal cancer
AP	acute pancreatitis
CEA	carcinoembryonic antigen
VEGF	vascular endothelial growth factor
EMT	epithelial-mesenchymal transition
EGFR	epidermal growth factor receptor
CCND1	cyclin D1
HCC	hepatocellular carcinoma
ITGβ4	Integrinβ4
MMP2	metalloproteinase 2
MMP9	metalloproteinase 9
BRAFi	BRAF inhibitor
PKC	Protein kinase C
MSTlR	c-Met-related tyrosine kinase
PRSS3	protease serine 3
PI3K	phosphatidylinositol 3-kinase
MAD2L2	Mitotic arrest deficient 2–like protein
CIN	chromosomal instability
CHD1L	chromodomain-helicase-DNA-binding-protein 1-like gene
CAP	cold atmospheric plasma
SN	silymarin nanoemulsion
SLCO1B3	The solute carrier organic anion transporter family member 1B3
CLM	CRC liver metastasis
PBS	phosphate-buffered saline
Bcl-2	B-cell lymphoma 2
TNF	the tumor necrosis factor
shRNA	short hairpin RNA
Bax	Bcl2 Associated X Protein
OS	overall survival
sPCL	secondary plasma cell leukaemia
SNVs	single nucleotide variations
hMSCs	human mesenchymal stem cells
TAC	transverse aortic constriction
PaCa	pancreatic cancer
LC3-II	microtubule-associated protein light chain 3-II
LAMP1	lysosomal-associated membrane protein 1
TFE3	the transcription factors E3
PD	Parkinson’s s disease
TH	Tyrosine Hydroxylase
p-JNK	phospho-c-Jun N-terminal Kinase
AKI	acute kidney injury
PIN	prostatic intraepithelial neoplasia
RPE	retinal pigment epithelial
MAPK	mitogen-activated protein kinase
AGS	acetaldehyde-generating system
